# Searching for Clinically Relevant Biomarkers in Geriatric Oncology

**DOI:** 10.1155/2018/3793154

**Published:** 2018-02-18

**Authors:** Theodora Katsila, George P. Patrinos, Dimitrios Kardamakis

**Affiliations:** ^1^Department of Pharmacy, School of Health Sciences, University of Patras, Patras, Greece; ^2^Department of Pathology, College of Medicine and Health Sciences, United Arab Emirates University, Al Ain, UAE; ^3^Department of Radiation Oncology, University of Patras Medical School, Patras, Greece

## Abstract

Ageing, which is associated with a progressive decline and functional deterioration in multiple organ systems, is highly heterogeneous, both inter- and intraindividually. For this, tailored-made theranostics and optimum patient stratification become fundamental, when decision-making in elderly patients is considered. In particular, when cancer incidence and cancer-related mortality and morbidity are taken into account, elderly patient care is a public health concern. In this review, we focus on oncogeriatrics and highlight current opportunities and challenges with an emphasis on the unmet need of clinically relevant biomarkers in elderly cancer patients. We performed a literature search on PubMed and Scopus databases for articles published in English between 2000 and 2017 coupled to text mining and analysis. Considering the top insights, we derived from our literature analysis that information knowledge needs to turn into knowledge growth in oncogeriatrics towards clinically relevant biomarkers, cost-effective practices, updated educational schemes for health professionals (in particular, geriatricians and oncologists), and awareness of ethical issues. We conclude with an interdisciplinary call to omics, geriatricians, oncologists, informatics, and policy-makers communities that* Big Data* should be translated into decision-making in the clinic.

## 1. Introduction

Age-related decline and functional deterioration in multiple organ systems are heterogeneous both inter- and intraindividually [1] and at times differ from the chronological age. To add further to this complexity, the mechanisms and molecular networks involved in the ageing process are still to be defined. Harman [2] stated that ageing occurs due to free radicals and radiation chemistry and several others have further supported this theory, according to which reactive oxygen species is the major determinant of lifespan, with an emphasis on protein oxidation [3] or mitochondria [4]. It has been also commonly assumed that growth and ageing share a common molecular mechanism, being the evolutionary conserved TOR (target of rapamycin) pathway [5]. To many, the oxidation-inflammation theory (“oxi-inflamm-ageing”) dominates, providing answers to the how (oxidation), where first (mitochondria of differentiated cells), and why (pleiotropic genes) this process occurs [6]. According to this integrative theory, the involvement of the immune system in “oxi-inflamm-ageing” is of paramount importance, since (i) the redox state relates to the functional capacity of immune cells and (ii) “immunosenescence” describes the age-associated failing systemic immunity [6, 7]. Others focus solely on “inflamm-ageing,” a chronic state of low-grade inflammation [8–10], “immunosenescence” [11, 12], or dysfunctional telomeres [13–15].

Taken together, the aforementioned theories and terms are often used to describe why cancer, infection, and autoimmune disease incidences all increase with age [16]. Another topic of debate refers to the age at which a patient is “elderly,” as it seems there is a lack of consensus across specialties and governing bodies [17, 18]. Thus, even though the generally accepted definition of elderly is that of being older than 65 years, many argue it should be older than 75 years, if the physiologic and pharmacologic changes that occur around that time are to be taken into account [19, 20].

**Figure 1:**
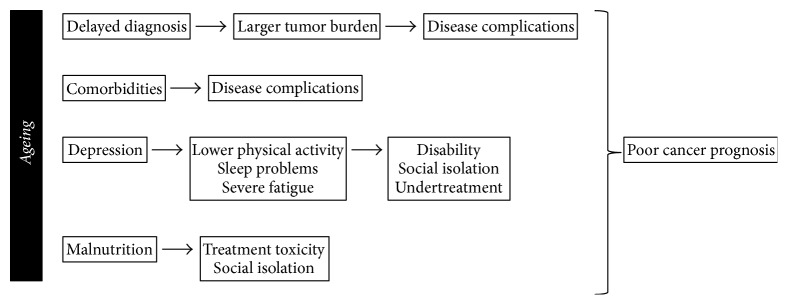
Cancer care in the elderly is of paramount importance as ageing itself is considered as the most important risk factor for cancer development and poor prognosis. Indeed, ageing has been associated with delayed diagnosis, comorbidities, depression, malnutrition, and, notably, undertreatment.

Whatever the mechanism(s) and network(s) underlying the ageing process may be or the criteria according to which a patient is defined as “elderly,” ageing itself is considered as the most important risk factor for cancer development and poor prognosis (Figure 1). To this extent, data from the National Cancer Institute Surveillance Epidemiology and End Results program reveals that the median age at diagnosis across all cancer types is 66 years, with more than 25% of new cancer cases being diagnosed in patients aged 65 to 74 years, 19% in those aged 75 to 84 years, and 8% in those older than 84 years (https://seer.cancer.gov/statfacts/html/all.html). As human population ages, an increasing cancer incidence is anticipated. If optimum cancer treatment is to be employed, then the life expectancy of cancer patients will be also greater.

As it becomes apparent, comprehensive geriatric assessment (CGA), being a biological assessment, still needs easily accessible biomarkers to predict the decline or loss of functional reserve. Such biomarkers should also enable their monitoring through various endpoints, such as overall survival or functional disability [21]. Especially for oncogeriatrics, clinically relevant biomarkers should (i) provide an accurate estimate of the general health status and disease-free or overall survival of patients, being independent of cancer-specific prognosis, (ii) evaluate the impact of comorbidities, and (iii) predict any treatment-related toxicities to inform decision-making towards optimum disease management. Today, decision-making is largely based on benefit/risk ratios and an assessment of the general health status of patients via the application of several algorithms [1] or in the context of adjuvant treatments survival estimates are derived from clinical scores [22, 23].

Herein, we performed a literature search on PubMed and Scopus databases for articles published in English between 2000 and 2017 coupled to text mining and analysis. We used MeSH and search terms (indicatively, “precision oncology”, “precision medicine”, “oncogeriatrics”, “biomarkers” and “theranostics”). Such terms were applied to “keyword,” “title,” and “abstract.” Our search strategy consisted of four phases (identification, screening, eligibility, and final inclusion) and has been further supported by HiPub [24] and FuseMind (https://fusemind.org). Our literature and text mining analyses highlight that information knowledge needs to turn into knowledge growth in oncogeriatrics. Considering that ageing is a rather complex trait, system-level multiomics strategies may unravel the complexity of oncogeriatrics and guide decision-making in geriatric oncology.

## 2. Candidate Biomarkers in Oncogeriatrics

### 2.1. Ageing Biomarkers

Ageing has a major impact on mitotically inactive organ systems (brain, heart) or their counterparts with high rates of cell turnover (haemopoietic and epithelial tissues) [25]. Thus, someone would expect that such organ systems offer a great repertoire of candidate ageing biomarkers. To name a few, the ageing of the hematopoietic system, which is one of the most studied and best characterized systems due to tissue accessibility by peripheral blood sampling, is marked by decrease in lymphopoiesis (often referred to by “immunosenescence” [11]) and a relative increase of myelopoiesis, possibly because of a selective depletion of lymphoid competent hematopoietic stem cells [12, 26]. Furthermore, the ageing associated decline in lymphopoiesis has been linked to dysfunctional telomeres. Dysfunctional telomeres not only are fundamental markers of cellular ageing, but also can induce inflammatory signals impairing lymphopoiesis [13–15, 25]. In the context of “inflamm-ageing,” several inflammation markers are detected in peripheral blood, whose increasing levels indicate the interplay of chronic inflammation and human ageing [27], an interplay which has been also described as being disturbed between autophagy and inflammasomes [28]. Indicatively, chronic high levels of C-reactive protein (CRP), tumor necrosis factor-alpha (TNF-alpha), and interleukin-6 (IL-6) levels have been shown to correlate with functional decline and survival [29]. Notably, the aforementioned markers depend on both ageing and inflammation, the latter being a pathobiological status. Questioning further their clinical relevance, Salvioli et al. [30] reappraised the concept of “inflamm-ageing,” implying that its pathological consequences can be independent of proinflammatory mediators and hence it is rather associated with the tissue and cell type in question. Monti et al. [31] presented an updated version of “inflamm-ageing” focused on glycomics that fully agrees with the extensive complexity that accompanies the interplay of “inflamm-ageing” and human longevity.

### 2.2. Clinically Relevant Ageing Biomarkers

**Figure 2:**
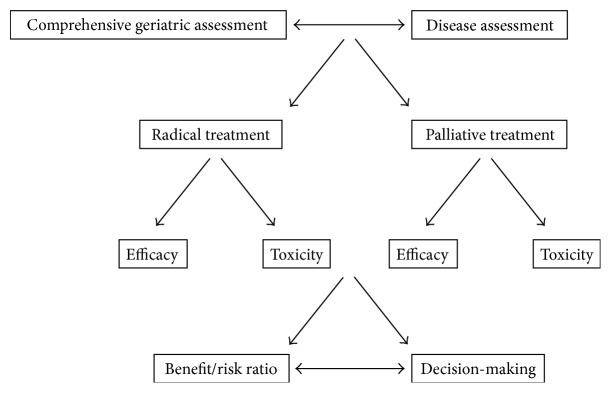
Oncogeriatrics holds the promise of informing decision-making in the clinic for the elderly cancer patients. In such a context, CGA (by geriatricians) coupled to disease assessment (by oncologists) will result in radical or palliative treatment strategies and, next, benefit/risk ratios (on the basis of efficacy/toxicity data) will guide decision-making. The role of clinically relevant ageing biomarkers is fundamental in every step of the decision-making process.

For clinically relevant ageing biomarkers to suffice for optimum decision-making and tailored-made theranostics in oncogeriatrics, they should (i) provide an accurate estimate of the general health status and disease-free or overall survival of patients, being independent of cancer-specific prognosis, (ii) evaluate the impact of comorbidities, and (iii) predict any treatment-related toxicities to inform decision-making towards optimum disease management (Figure 2). The ageing process not only affects cancer strategies and patient stratification in light of related toxicities and theranostics' inefficacy, but also may be also affected by the disease state itself. An increasing amount of data indicates that the accumulation of damaged cells may influence the rate of ageing as well as the development of ageing associated diseases in cancer patients, as the general health status of cancer survivors is compromised when compared to that of the general population [39–41].

Table 1 summarizes candidate biomarkers in oncogeriatrics and points out as primary interest (i) DNA damage (upstream) markers, (ii) DNA damage induced alterations in tissue composition (e.g., immune system), (iii) the induction of cell senescence, (iv) senescent-associated secretory alterations, and (v) telomere dysfunction. Yet, age-related alterations that are central to cancer development and hence may serve as clinically relevant biomarkers are highly complex and as such they remain an area of active study. Other key contributing factors include somatic mutational load and epigenetic regulation as well as a changing stromal environment [16, 42].

Since the ability of the elderly patients to tolerate treatment and the overall benefit/risk ratio are important parameters to be taken into account in decision-making in oncogeriatrics, clinically relevant biomarkers are also needed to assess risk factors and predict efficacy/toxicity upon xenobiotic administration. Current data are scarce, since specific efficacy and safety data by age in clinical trials are extremely limited. The case of candidate biomarkers of response when elderly cancer patients are treated with immunotherapy strategies serves as a paradigm. Indeed, Meucci et al. have explored a head and neck squamous cell carcinoma cohort of the Cancer Genome Atlas and reported age-dependent differences in mutational backgrounds of tumors with four specific enriched pathways (namely, “axon guidance,” “focal adhesion,” “ECM-receptor interaction,” and “Notch signalling”) that were only sporadically mutated in the other age groups [43].

Even though such findings would suggest that older patients should be more likely to respond to immune checkpoint blockade, this has not been documented clearly in clinical trials to date [16]. Talarico et al. [44] analysed for the first time the age-related enrolment of cancer patients onto registration trials of either new drugs or indications approved by the US Food and Drug Administration (1995–2002), concluding that elderly cancer patients were underrepresented (the percentages of study patients aged ≥65, 70, and 75 years were compared with the corresponding percentages in the US cancer population for the treatment of leukaemia, lymphoma, and cancers of the breast, lung, colon or rectum, ovary, pancreas, and central nervous system).

Earlier this year, Bailur et al. [45] conducted the first clinical study to identify biological and clinical ageing biomarkers in elderly breast cancer patients receiving chemotherapy, integrating geriatric assessment data to blood-based candidate biomarkers (leukocyte telomere length, plasma cytokines, and growth factors), circulating microRNAs (miRNAs), and immune (cytomegalovirus serostatus, circulating immune cell populations) measurements. Notably, alterations in immune profiles over time were obtained coupled to specific circulating leukocyte populations measured prior to therapy (elevated CD4+ T effector memory reexpressing CD45RA cells and relatively lower CD8+ central memory cells at 3 months, with normalized levels after 12 months) and biomarkers of ageing, including telomere length and blood cytokines, and clinical frailty scored by the LOFS (Leuven Oncogeriatric Frailty Score) [46] and G8 screening tool [47]. An interesting finding is that none of the immune populations studied correlated with chronological age, while immune profiles prior to therapy predicted unexpected hospitalizations in patients receiving chemotherapy. As the authors point out, however, this study was exploratory and thus no power calculations were performed or correction for multiple testing for a large number of the statistical tests performed.

In particular for circulating miRNAs, when Hatse et al. [48] determined miRNA expression levels (175 plasma/serum miRNAs) in elderly breast cancer patients (*n* = 10) upon chemotherapy administration to identify “ageing miRNAs” for monitoring the impact of the therapeutic approach on their biological age, the age-associated miRNAs did not show differential expression between fit/healthy and nonfit/frail subjects within the elderly breast cancer cohort of the validation study (*n* = 10 fit and *n* = 10 frail patients).

## 3. From Precision Oncology to Geriatric Oncology (Oncogeriatrics)

Precision oncology holds the promise of more-effective, less-toxic tailored-made theranostics as well as optimum patient stratification therapies on the basis of genomic, molecular, or related characteristics of cancers that can shape treatment or elucidate prognosis [49]. Experiencing the* Big Data* era, notwithstanding, such information growth needs to be curated, disseminated, and translated into knowledge growth for the medical oncologist to provide precision oncology and value-based care to cancer patients.

For the elderly patients with cancer, geriatric oncology holds such promise, as it is well established that cancer is a disease state that peaks after 60 years of age and human populations are inevitably ageing. Indeed, cancer-related mortality and morbidity in the elderly are a public health concern [50]. Yet, the routine CGA for the management of elderly patients with cancer still calls upon the skills of both oncologists and geriatricians, as the former should not perform geriatric assessments themselves, nor should geriatricians recommend cancer treatment strategies [51]. For this, medical students and health professionals need to be educated.

Even though the progress in the understanding of human biology has been tremendous, the mechanisms and networks driving ageing remain largely unresolved and at the same time geriatric patients are a highly heterogeneous group for which ageing related alterations cannot be delineated on the basis of chronological age alone. In this context, optimum patient care becomes rather challenging, even when CGA is considered, as it is a time-consuming staff-intensive procedure. Therefore, user-friendly cost-effective tools are critical in everyday clinical practice. Spyropoulou et al. [52] explored the use of the Vulnerable Elders Survey-13 (VES-13) score [53] as an accurate predictive tool towards optimum patient stratification, assessing the vulnerability of elderly patients with cancer and planning radiotherapy in a case-specific manner. The authors demonstrated for the first time that patients with higher VES-13 scores were highly likely not to complete radiotherapy and such an association was found to be independent of confounding factors (age, sex, comorbidities, toxicity, and type of radiotherapy). Such tools can be further empowered by web-based informatics interfaces, ideally coupled to electronic health records that could be also transferable to different clinical settings.

Exploring further approaches to address the complexity and challenges in managing elderly patients with cancer, clinicians need to be introduced to and use CGA omics data. To structure medical decisions in face of uncertainty, information needs to be translated into clinically relevant knowledge and this is the exact advantage that CGA omics data offer, especially if coupled to user-friendly informatics. Recently, clinical cases have served as paradigms to show the interoperability of an algorithm for optimum decision-making in elderly patients with cancer, focusing on the assessment of (i) cancer-free life expectancy, (ii) cancer-related risks on patient survival, function, or quality of life, and (iii) treatment-related risks and benefits [54]. Notably and as it has been recently showed by a systematic review of systematic reviews with a focus on the use of CGA tools to predict adverse postoperative outcomes [55], different clinical settings present a range of demands and needs, when daily clinical practice is taken into account. For this, CGA omics data may serve as the knowledge platform to tailor a transferable geriatric assessment across various clinical settings and practices. CGA omics data are* Big Data* and if stringent criteria are employed, robustness and feasibility can be supported. In this context and if companion theranostics are considered, disease and response/toxicity biomarker discovery and validation are crucial. Today, clinically relevant ageing biomarkers remain a matter of debate, as neither cross-sectional nor longitudinal studies could emerge any biomarkers usable in clinical studies on ageing [21, 56]. Once again,* Big Data* coupled to evidence-based stringent criteria and informatics may pave the way to informed biomarker discovery and validation.

There is no doubt that clinical trials with larger accrual of cancer patients, and elderly patients in particular, are critical for the assessment of the efficacy and safety of theranostics. Elderly patients are still underrepresented in clinical trials, rendering evidence-based decision-making for the elderly patients challenging. Interestingly, there are race-, sex-, and age-based disparities to be considered, which remain a matter of debate regarding participation in cancer clinical trials [57, 58]. Furthermore, a consensus should be reached regarding the definition of a patient/individual as “elderly” [17, 18].

In terms of general health policies, cost-effective oncogeriatric practices need to be demonstrated, along with the evidence that if the adequate cancer care strategy with the help of geriatric assessment is chosen, then positive outcomes with minimal toxicity are to be obtained for the elderly cancer patients deemed fit for treatment. At this point, ethical issues arise, especially when frail patients are considered, since it is deemed best for them to undergo best supportive care rather than radical therapy [59]. Furthermore, in the current antiageism era, we must consider the putative harmful consequences of the use of ageing biomarkers [21].

## 4. Conclusions

As the human population increases and ages with time, tailored-made theranostics for the elderly become of paramount importance. When cancer incidence is considered, cancer-related mortality and morbidity in the elderly become an immediate public health concern. Clinically ageing biomarkers could become a quantitative, reproducible, and quick help for decision-making in the clinic. Even though data are still sparse and the current challenges are many, oncogeriatrics holds the promise of more-effective, less-toxic tailored-made theranostics as well as optimum patient stratification therapies on the basis of genomic, molecular, or related characteristics of cancers that can shape treatment or elucidate prognosis in the elderly. In such a context, clinicians need to be introduced to and use CGA omics data coupled to user-friendly informatics.

## Figures and Tables

**Table 1:** Candidate disease biomarkers in oncogeriatrics.

Biological process	Candidate disease biomarkers	Refs
DNA damage (upstream)	Gamma-H2AX, 53BP1, MDC1	[21, 32]

DNA damage induced alterations in tissue composition (e.g., immune system)	Decreased production of naïve lymphocytes with a concomitant increase of myeloid cell lineages	[16, 21, 32]

Senescence induction	p21, p16, and SA-beta-GAL	[21, 33]

Senescence-associated secretory alterations	G-SCF, IL-6, IL-8, GRO (a, b, g), IL-7, ICAM-1	[21, 32, 34, 35]

Telomere dysfunction	Telomere shortening, altered expression of proteins of the shelterin, anaphase bridges, and chromosomal imbalances	[21, 36–38]

## References

[1] Balducci L., Extermann M. (2000). Management of cancer in the older person: A practical approach. *The Oncologist*.

[2] Harman D. (1956). Aging: a theory based on free radical and radiation chemistry. *Journal of Gerontology*.

[3] Stadtman E. R. (1992). Protein oxidation and aging. *Science*.

[4] Balaban R. S., Nemoto S., Finkel T. (2005). Mitochondria, oxidants, and aging. *Cell*.

[5] Blagosklonny M. V., Hall M. N. (2009). Growth and aging: a common molecular mechanism. *Aging*.

[6] de La Fuente M., Miquel J. (2009). An update of the oxidation-inflammation theory of aging: the involvement of the immune system in Oxi-Inflamm-Aging. *Current Pharmaceutical Design*.

[7] Pawelec G., Larbi A., Derhovanessian E. (2010). Senescence of the Human Immune System. *Journal of Comparative Pathology*.

[8] Franceschi C., Bonafè M., Valensin S. (2000). Inflamm-aging. An evolutionary perspective on immunosenescence. *Annals of the New York Academy of Sciences*.

[9] Baylis D., Bartlett D. B., Patel H. P., Roberts H. C. (2013). Understanding how we age: insights into inflammaging. *Longevity & Healthspan*.

[10] Franceschi C., Campisi J. (2014). Chronic inflammation (inflammaging) and its potential contribution to age-associated diseases. *The Journals of Gerontology. Series A, Biological Sciences and Medical Sciences*.

[11] Wang J., Geiger H., Rudolph K. L. (2011). Immunoaging induced by hematopoietic stem cell aging. *Current Opinion in Immunology*.

[12] Wang J., Sun Q., Morita Y. (2012). A differentiation checkpoint limits hematopoietic stem cell self-renewal in response to DNA damage. *Cell*.

[13] Song Z., Wang J., Guachalla L. M. (2010). Alterations of the systemic environment are the primary cause of impaired B and T lymphopoiesis in telomere-dysfunctional mice. *Blood*.

[14] Song Z., Zhang J., Ju Z., Rudolph K. L. (2012). Telomere dysfunctional environment induces loss of quiescence and inherent impairments of hematopoietic stem cell function. *Aging Cell*.

[15] Von Figura G., Hartmann D., Song Z., Rudolph K. L. (2009). Role of telomere dysfunction in aging and its detection by biomarkers. *Journal of Molecular Medicine*.

[16] Marrone K. A., Forde P. M. (2017). Cancer immunotherapy in older patients. *Cancer Journal (United States)*.

[17] Levine M. E. (2013). Modeling the rate of senescence: can estimated biological age predict mortality more accurately than chronological age?. *The Journals of Gerontology. Series A, Biological Sciences and Medical Sciences*.

[18] Singh S., Bajorek B. (2014). Defining ‘elderly’ in clinical practice guidelines for pharmacotherapy. *Pharmacy Practice*.

[19] McLean A. J., le Couteur D. G. (2004). Aging biology and geriatric clinical pharmacology. *Pharmacological Reviews*.

[20] Orimo H., Ito H., Suzuki T., Sawabe M. (2006). Reviewing the definition of “elderly”. *Geriatrics & Gerontology International*.

[21] Falandry C., Gilson E., Rudolph K. L. (2013). Are aging biomarkers clinically relevant in oncogeriatrics?. *Critical Review in Oncology/Hematology*.

[22] Lee S. J., Lindquist K., Segal M. R. (2006). Development and Validation of a Prognostic Index for 4-Year Mortality in Older Adults. *Journal of the American Medical Association*.

[23] Yourman L. C., Lee S. J., Schonberg M. A., Widera E. W., Smith A. K. (2012). Prognostic indices for older adults: A systematic review. *Journal of the American Medical Association*.

[24] Lee K., Shin W., Kim B. (2016). HiPub: Translating PubMed and PMC texts to networks for knowledge discovery. *Bioinformatics*.

[25] Falandry C., Bonnefoy M., Freyer G., Gilson E. (2014). Biology of cancer and aging: A complex association with cellular senescence. *Journal of Clinical Oncology*.

[26] Sieburg H. B., Cho R. H., Dykstra B., Uchida N., Eaves C. J., Muller-Sieburg C. E. (2006). The hematopoietic stem compartment consists of a limited number of discrete stem cell subsets. *Blood*.

[27] Goto M. (2008). Inflammaging (inflammation + aging): a driving force for human aging based on an evolutionarily antagonistic pleiotropy theory?. *Bioscience Trends*.

[28] Salminen A., Kaarniranta K., Kauppinen A. (2012). Inflammaging: disturbed interplay between autophagy and inflammasomes. *AGING*.

[29] Giovannini S., Onder G., Liperoti R. (2011). Interleukin-6, C-reactive protein, and tumor necrosis factor-alpha as predictors of mortality in frail, community-living elderly individuals. *Journal of the American Geriatrics Society*.

[30] Salvioli S., Monti D., Lanzarini C. (2013). Immune system, cell senescence, aging and longevity - inflamm-aging reappraised. *Current Pharmaceutical Design*.

[31] Monti D., Ostan R., Borelli V., Castellani G., Franceschi C. (2017). Inflammaging and human longevity in the omics era. *Mechanisms of Ageing and Development*.

[32] Hubbard J. M., Cohen H. J., Muss H. B. (2014). Incorporating biomarkers into cancer and aging research. *Journal of Clinical Oncology*.

[33] Wadhwa R., Kaul Z., Kaul S. C. (2016). Cell Cycle Checkpoints and Senescence. *Cellular Ageing and Replicative Senescence*.

[34] Coppé J.-P., Patil C. K., Rodier F. (2008). Senescence-associated secretory phenotypes reveal cell-nonautonomous functions of oncogenic RAS and the p53 tumor suppressor. *PLoS Biology*.

[35] Kowdley G. C., Merchant N., Richardson J. P., Somerville J., Gorospe M., Cunningham S. C. (2012). Cancer surgery in the elderly. *The Scientific World Journal*.

[36] Ju Z., Jiang H., Jaworski M. (2007). Telomere dysfunction induces environmental alterations limiting hematopoietic stem cell function and engraftment. *Nature Medicine*.

[37] Falandry C., Horard B., Bruyas A. (2015). Telomere length is a prognostic biomarker in elderly advanced ovarian cancer patients: A multicenter GINECO study. *AGING*.

[38] Li D., de Glas N. A., Hurria A. (2016). Cancer and Aging: General Principles, Biology, and Geriatric Assessment.. *Clinics in Geriatric Medicine*.

[39] Prasad P. K., Signorello L. B., Friedman D. L., Boice J. D., Pukkala E. (2012). Long-term non-cancer mortality in pediatric and young adult cancer survivors in Finland. *Pediatric Blood & Cancer*.

[40] Hammerlid E., Taft C. (2001). Health-related quality of life in long-term head and neck cancer survivors: A comparison with general population norms. *British Journal of Cancer*.

[41] Cella D., Lai J.-S., Chang C.-H., Peterman A., Slavin M. (2002). Fatigue in cancer patients compared with fatigue in the general United States population. *Cancer*.

[42] DePinho R. A. (2000). The age of cancer. *Nature*.

[43] Meucci S., Keilholz U., Tinhofer I., Ebner O. A. (2016). Mutational load and mutational patterns in relation to age in head and neck cancer. *Oncotarget *.

[44] Talarico L., Chen G., Pazdur R. (2004). Enrollment of elderly patients in clinical trials for cancer drug registration: A 7-year experience by the US Food and Drug Administration. *Journal of Clinical Oncology*.

[45] Bailur J. K., Pawelec G., Hatse S. (2017). Immune profiles of elderly breast cancer patients are altered by chemotherapy and relate to clinical frailty. *Breast cancer research: BCR*.

[46] Brouwers B., Dalmasso B., Hatse S. (2015). Biological ageing and frailty markers in breast cancer patients. *AGING*.

[47] Soubeyran P., Bellera C., Goyard J. (2011). Validation of the G8 screening tool in geriatric oncology: The ONCODAGE project. *Journal of Clinical Oncology*.

[48] Hatse S., Brouwers B., Dalmasso B. (2014). Circulating MicroRNAs as easy-to-measure aging biomarkers in older breast cancer patients: Correlation with chronological age but not with fitness/frailty status. *PLoS ONE*.

[49] Hughes K. S., Ambinder E. P., Hess G. P. (2017). Identifying health information technology needs of oncologists to facilitate the adoption of genomic medicine: Recommendations from the 2016 American society of clinical oncology omics and precision Oncology workshop. *Journal of Clinical Oncology*.

[50] Kurtz J.-E., Heitz D., Kurtz-Illig V., Dufour P. (2009). Geriatric oncology: How far have we gone and what are the next steps?. *Oncology*.

[51] Kurtz J.-E., Heitz D., Enderlin P. (2010). Geriatric oncology, general practitioners and specialists: Current opinions and unmet needs. *Critical Review in Oncology/Hematology*.

[52] Spyropoulou D., Pallis A. G., Leotsinidis M., Kardamakis D. (2014). Completion of radiotherapy is associated with the Vulnerable Elders Survey-13 score in elderly patients with cancer. *Journal of Geriatric Oncology*.

[53] Saliba D., Elliott M., Rubenstein L. Z. (2001). The vulnerable elders survey: a tool for identifying vulnerable older people in the community. *Journal of the American Geriatrics Society*.

[54] Vallet-Regí M., Manzano M., Rodriguez-Mañas L., López M. C., Aapro M., Balducci L. (2017). Management of cancer in the older age person: An approach to complex medical decisions. *The Oncologist*.

[55] Huisman M. G., Kok M., de Bock G. H., van Leeuwen B. L. (2017). Delivering tailored surgery to older cancer patients: Preoperative geriatric assessment domains and screening tools – A systematic review of systematic reviews. *European Journal of Surgical Oncology*.

[56] Hurria A., Jones L., Muss H. B. (2016). Cancer Treatment as an Accelerated Aging Process: Assessment, Biomarkers, and Interventions. *American Society of Clinical Oncology educational book. American Society of Clinical Oncology. Meeting*.

[57] Kemeny M. M., Peterson B. L., Kornblith A. B. (2003). Barriers to clinical trial participation by older women with breast cancer. *Journal of Clinical Oncology*.

[58] Murthy V. H., Krumholz H. M., Gross C. P. (2004). Participation in cancer clinical trials: Race-, sex-, and age-based disparities. *Journal of the American Medical Association*.

[59] Basso U., Tonti S., Bassi C. (2008). Management of Frail and Not-Frail elderly cancer patients in a hospital-based geriatric oncology program. *Critical Review in Oncology/Hematology*.

